# A targeted liquid cultivation method for previously uncultured non-colony forming microbes

**DOI:** 10.3389/fmicb.2023.1194466

**Published:** 2023-06-09

**Authors:** Eun-Young Seo, Dawoon Jung, Slava S. Epstein, Weiyan Zhang, Jeffrey S. Owen, Hiroaki Baba, Akina Yamamoto, Mifuyu Harada, Yutaka Nakashimada, Setsu Kato, Yoshiteru Aoi, Shan He

**Affiliations:** ^1^Li Dak Sum Yip Yio Chin Kenneth Li Marine Biopharmaceutical Research Center, College of Food and Pharmaceutical Sciences, Ningbo University, Ningbo, China; ^2^Ningbo Institute of Marine Medicine, Peking University, Ningbo, China; ^3^Department of Molecular Biotechnology, Graduate School of Advanced Sciences of Matter, Hiroshima University, Higashihiroshima, Japan; ^4^Department of Biology, Northeastern University, Boston, MA, United States; ^5^Department of Environmental Science, Hankuk University of Foreign Studies, Yongin, Republic of Korea; ^6^Graduate School of Integrated Sciences for Life, Hiroshima University, Higashihiroshima, Japan

**Keywords:** non-colony-forming, liquid cultivation, dilution-to-extinction, uncultured microbes, microbial dark matter

## Abstract

A large number of microbes are not able to form colonies using agar-plating methods, which is one of the reasons that cultivation based on solid media leaves the majority of microbial diversity in the environment inaccessible. We developed a new Non-Colony-Forming Liquid Cultivation method (NCFLC) that can selectively isolate non-colony-forming microbes that exclusively grow in liquid culture. The NCFLC method involves physically separating cells using dilution-to-extinction (DTE) cultivation and then selecting those that could not grow on a solid medium. The NCFLC was applied to marine samples from a coastal intertidal zone and soil samples from a forest area, and the results were compared with those from the standard direct plating method (SDP). The NCFLC yielded fastidious bacteria from marine samples such as *Acidobacteriota, Epsilonproteobacteria, Oligoflexia*, and *Verrucomicrobiota*. Furthermore, 62% of the isolated strains were potential new species, whereas only 10% were novel species from SDP. From soil samples, isolates belonging to *Acidobacteriota* and *Armatimonadota* (which are known as rare species among identified isolates) were exclusively isolated by NCFLC. Colony formation capabilities of isolates cultivated by NCFLC were tested using solid agar plates, among which approximately one-third of the isolates were non-colony-forming, approximately half-formed micro-colonies, and only a minority could form ordinary size colonies. This indicates that the majority of the strains cultivated by NCFLC were previously uncultured microbial species unavailable using the SDP method. The NCFCL method described here can serve as a new approach to accessing the hidden microbial dark matter.

## 1. Introduction

Most microbes in the environment are still uncultured and have been referred to as the “microbial dark matter” (Rinke et al., 2013; Lok, 2015). Only a few microbes (generally about 1%) can grow into colonies on standard solid agar media, thus leading to a “great plate count anomaly” first noted more than 120 years ago (Winterberg, 1898; Staley and Konopka, 1985; Amann et al., 1995). This unresolved puzzle in microbiology remains the major bottleneck in bioprospecting for microbial resources (Jiao et al., 2021).

Despite the availability of culture-independent molecular tools for the analysis of microbial communities (Wang et al., 2012) and heterologous expression of biosynthetic gene clusters (Ongley et al., 2013; Gomez-Escribano and Bibb, 2014; Nah et al., 2017), culture-dependent approaches are still necessary because having comprehensive knowledge of physiological characteristics and improving the potential for various biotechnological applications essentially requires isolating individual bacterial strains in pure culture (Vartoukian et al., 2010).

The mechanisms explaining why most microbes cannot grow using conventional cultivation methods are still unclear. Studies on novel cultivation approaches have recommended various methods that led to the isolation of some previously uncultured microbial species (Lewis et al., 2021), including membrane diffusion-based cultivation for *in situ* cultivation, such as the isolation chip (Bollmann et al., 2007; Berdy et al., 2017), hollow-fiber membrane chambers (HFMC) (Aoi et al., 2009), diffusion bioreactors (Chaudhary et al., 2019), and the soil substrate membrane system (SSMS) (Svenning et al., 2003; Ferrari et al., 2005). These methods use naturally occurring growth factors to better reflect what is available to microbes in their own habitat. Microfluidic-based cultivation methods such as nanoporous microscale microbial incubators (NMMI) (Ge et al., 2016), chemotactic sorting with droplet microfluidics (Dong et al., 2016), or the SlipChip (Du et al., 2009; Ma et al., 2014) manipulate cells in small volumes and with large numbers of replicates, as well as the cell sorting-based techniques, such as Raman-activated cell sorting (RACS) (Lau et al., 2008; Lee et al., 2019), fluorescence *in situ* hybridization of live cells (live-FISH) (Batani et al., 2019), and reverse genomics (Cross et al., 2019), have all targeted functional or taxonomic subsets of cells for isolation. However, the majority of extant microbes in the environment, especially from marine habitats, remain uncultivated. Thus, there must be hidden factors impeding microbial cultivation that have yet to be identified.

Non-colony-forming microbes feature a microbial type for which it is impossible or difficult to cultivate colonies that are visible to the naked eye on solid agar plates using microbial cultivation and isolation procedures. Previous studies have demonstrated that non-colony-forming bacteria constituted the majority of viable bacterial cells and made up a significant fraction of marine bacterioplankton (Giovannoni and Rappé, 2000; Simu and Hagström, 2004). Eilers et al. (2000) and Simu et al. (2005) found that non-colony-forming bacteria are abundant, sometimes dominant, in pelagic bacterial communities. In our previous study, we demonstrated that long-term liquid cultivation using a continuous-flow bioreactor led to cultivating previously uncultured bacteria from marine sponges (Jung et al., 2022). Interestingly, physiological analysis of bacterial species isolated using the bioreactor showed that these species had difficulty forming colonies. Specifically, this bacterial type can be cultivated and enriched in a liquid medium but not on standard agar plates. Therefore, we assumed that a significant number of non-colony-forming bacteria exist in sponge tissue and other marine environments, which partially explains the long-lasting problem of accessing hidden environmental microbial diversity.

The dilution-to-extinction cultivation method was developed and successfully applied for the cultivation of microbial groups that are the most abundant in marine environments but was previously uncultured (Button et al., 1998; Giovannoni and Rappé, 2000; Connon and Giovannoni, 2002; Cho and Giovannoni, 2004). However, it is not only ideally compatible with liquid cultivation but also provides a physical barrier between isolates to avoid their faster growth by taking into account all situations found in conventional methods, since non-colony-forming bacteria are usually slow growers (Olsen and Bakken, 1987; Kato et al., 2018).

Therefore, we developed a new Non-Colony-Forming Liquid Cultivation (NCFLC) method that targets non-colony-forming microbes that exclusively grow in liquid culture. As a proof of concept, we applied the new liquid cultivation method to different types of intertidal marine samples to selectively isolate previously uncultured microbes. The NCFLC method was also applied to soil samples to test if the new cultivation method was effective for samples from a terrestrial environment. In addition, we identified some characteristics of the isolates to elucidate possible mechanisms operating in NCFLC.

## 2. Material and methods

### 2.1. Sample collection

Three types of samples from the intertidal zone—sediment, seawater, and marine plant roots—were collected on the northwest coast of Meishan Island, Ningbo, China. The sediment and seawater samples were collected in an area without macrophytes (GPS coordinates: N29°45′51.89″, E121°55′42.97″) between September and November 2019. The surface sediment (1–3 cm depth) and seawater samples were collected at the same site in sterile conical tubes. The roots were collected in an area with macrophytes (GPS coordinates: N29°47′36.55″, E121°57′46.32″) in December 2020. The reeds (*Phragmites australis*) were harvested from the intertidal zone, and the roots were collected in a sterile bag after the other parts were physically separated. The salinity of the sampling sites, without macrophytes and with macrophytes, was 18.7 and 19.5‰, respectively. Samples were transported immediately to the laboratory for further culture-dependent and culture-independent experiments. Depending on the sample type, one (plant roots) or two replicates (sediment and seawater) were used. The soil samples were collected from a forest area inside the Hiroshima University campus, Higashihiroshima, Japan (GPS coordinates: N34°40′08.43″, E132°71′24.09″) between June 2018 and April 2021. The surface soil was collected from the uppermost layer (2–5 cm depth) in a sterile bag and transported immediately to the laboratory for cultivation experiments.

### 2.2. Media preparation

For the sediment, seawater, and marine plant roots, we used the following three media: (1) 1:2 diluted Marine Broth 2216 (50% of manufacturer's suggested concentration, Difco, Franklin Lakes, NJ, USA); (2) 1:10 diluted R2A Broth (10% of the manufacturer's suggested concentration, Hopebio, Qingdao, China); and (3) 1:100 diluted Nutrient Broth (1% of the manufacturer's suggested concentration, Hopebio, Qingdao, China). All media were supplemented with additional artificial sea salt (Instant Ocean, USA) to a final concentration of 2%. For the soil sample, we used one medium, 1:10 diluted R2A Broth (10% of the manufacturer's suggested concentration, Nihon Seiyaku, Japan).

### 2.3. Targeted liquid cultivation method for non-colony-forming microbes

**Figure 1:**
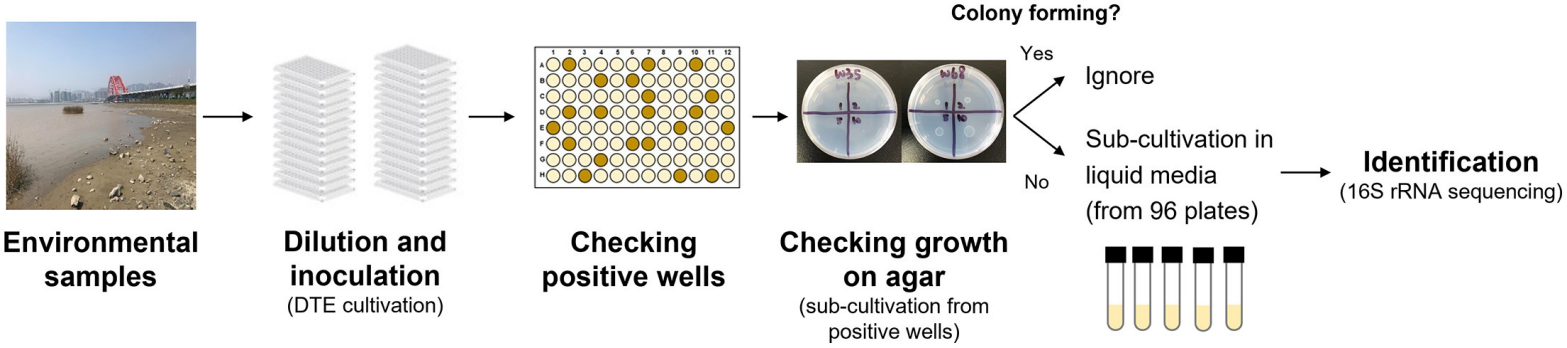
Experimental procedures in the NCFLC method.

To develop a new cultivation method targeting the bacterial type that cannot grow on agar plates, we developed a novel procedure termed Non-Colony-Forming Liquid Cultivation (NCFLC; Figure 1). To prepare the inoculum, 1 g of the sediment sample was placed into a 15 ml conical tube with 10 ml of sterile artificial seawater (2%) and vortexed for 10 min. The seawater sample was used directly as a source of inoculum. For root samples, ~5 g (wet weight) of the sample was gently rinsed twice with sterile artificial seawater (2%) to remove microbes that were not associated with the plants. Washed roots were cut into fragments of 0.5 cm in length, transferred into 50 ml conical tubes containing sterile artificial seawater (2%), and vortexed for 1 h. For all samples, any debris in the treated suspensions was removed by centrifugation at 1,500 rpm for 10 min.

For soil samples, 1 g of soil was placed into a 15 ml conical tube with 10 ml of 10% sterile phosphate-buffered saline (PBS) and vortexed for 10 min. Debris in the treated suspensions was removed by centrifugation at 1,500 rpm for 10 min followed by filtration with a nylon net filter (30 μm pore size; Merck-Millipore, USA), a polycarbonate membrane filter (10 μm pore size; ADVANTEC, Japan), and a mixed cellulose ester membrane filter (5 μm pore size; ADVANTEC, Japan).

First, we used dilution-to-extinction (DTE) cultivation in 96-well plates (Jung et al., 2021a). Pre-treated samples were diluted 10^−5^ to 10^−8^ with sterile artificial seawater (2%), and 200 μl of each dilution was then dispensed into a 96-well clear flat-bottom polystyrene microplate [VIOLAMO (ASONE), Osaka, Japan]. We prepared multiple plates as a dilution series, with each dilution made in triplicate. After incubation at 25°C for 4 weeks, cellular growth in each well was scored visually (roughly OD660 nm >0.05). Multi-well plates showing 10%−50% positive wells per plate were selected as the optimal dilution and used for sub-cultivation. Next, 5 μl of cell suspension from each positive well was inoculated on agar plates supplemented with the same media used in liquid cultivation. After 4 weeks of incubation, colony formation was examined using an optical microscope (Digital microscope, Hyrox, Tokyo, Japan). We selected and counted the positive wells that showed cell growth in multi-well plates but did not show colony formation at the sub-cultivation step. We presumed that the bacterial cultures in the wells were candidate strains that had difficulty growing on agar plates. Selected cultures were sub-cultivated in fresh liquid medium (5 ml) in test tubes and used for taxonomic identification. Approximately 60 bacterial isolates were isolated and identified for each sample from marine environments, and 93 bacterial isolates were identified from the soil samples.

### 2.4. Standard direct plating cultivation

To confirm the efficiency of the NCFLC method, the SDP method was used simultaneously as a control. The SDP method employed the same inoculant and media (1.5% agar) used for the liquid cultivation method. Samples were serially diluted, and each 50 μl of the samples was inoculated on an agar plate (1.5%) following the steps described in the “Media preparation” section. The plates were incubated for 4 weeks at room temperature, with 60 colonies per sample for marine samples and 203 colonies for the soil samples randomly selected and further purified for microbial identification.

### 2.5. Identification of isolates based on 16S rRNA sequencing

Taxonomic identification was performed by sequencing ~750–800 bp long fragments of the 16S rRNA gene for isolates from the samples. To identify isolates obtained from the NCFLC method, aliquots (1 ml) were centrifuged at 5,000 rpm at room temperature for 10 min. The supernatant was discarded, and the bacterial cells were collected as pellets. Pellets were washed three times by re-suspending with 0.5 ml DNA-free water, centrifuged at 5,000 rpm at room temperature for 10 min, and the supernatant was discarded. The purified cells were used as a template for PCR. For the identification of isolates obtained by SDP, colonies were used directly as a template for PCR. The universal primers 27F (5′-AGAGTTTGATCCTGGCTCAG-3′) and 1492R (5′-GGTTACCTTGTTACGACTT-3′) were used to amplify the 16S rRNA gene with a PCR system recommended by the manufacturer (Sangon Biotech, China). The PCR products were sequenced commercially (Sangon Biotech, China) by fluorescent dye terminator sequencing. The sequence results were determined at www.ezbiocloud.net/ (Yoon et al., 2017). Distance matrices and phylogenetic trees based on 16S rRNA sequences were built, and operational taxonomic units (OTUs) with ≥97% 16S rRNA gene sequence were identified, according to the Kimura two-parameter model and neighbor-joining algorithms using the MEGA 11 program (MEGA software, Tempe, AZ, USA). Newly determined sequence data have been deposited in GenBank (www.ncbi.nlm.nih.gov) under accession numbers (Supplementary Tables S1, S3, S4).

For isolates from the soil sample, taxonomic identification was performed by sequencing ~600–650 bp long fragments of the 16S rRNA gene. To identify isolates from NCFLC, aliquots (1 ml) were centrifuged at 5,000 rpm at room temperature for 10 min. The supernatant was discarded, followed by adding the same volume (900 μl) of sterile DNA-free water to resuspend the bacterial cell pellets. This step was repeated twice. Finally, the pellets were resuspended with 100 μl of DNA-free water and were used as a template for PCR. The 16S rRNA gene was amplified using the universal primers 27F and 1492R, with a KOD FX Neo system (Toyobo, Osaka, Japan), and then purified using a PCR products purification kit (AMPureXP, Beckman Coulter, CA, USA). The purified PCR products were sequenced commercially (Takara Bio, Shiga, Japan) by fluorescent dye terminator sequencing.

### 2.6. Growth characteristics of isolated strains

Growth curves of the isolates from the soil samples were analyzed to compare bacterial growth among the strains from each cultivation method. For further investigation, we selected 17 strains obtained from the NCFLC method, representing all OTUs considered as the bacterial type that can only grow in liquid (non-colony-forming type; eight strains) and nine randomly selected strains from the micro-colony-forming type. Non-colony-forming type strains did not show detectable growth on agar plates. In contrast, the micro-colony-forming type strains showed some growth on agar plates. However, they did not form visible isolated colonies observed by the naked eye, which can only be observed using a dissecting microscope. We also randomly selected 27 strains from SDP to compare the results. The R2A medium (5 ml) was inoculated with 5–20 μl of cell suspension, in triplicate, from the grown pure liquid cultures, and then incubated at 25°C with shaking at 120 rpm. A few strains did not grow with the R2A medium. In this case, the 1:10 diluted R2A medium was used. The optical density (OD) was measured at 600 nm using a spectrophotometer (DR 3900, HACH, USA) or a non-contact turbidimeter for cultures (OD-Monitor C&T, TAITEC Corporation, Saitama, Japan). To test some strains with aggregated growth that made it impossible to evaluate the growth characteristic from OD values, medium bottles (containing 100 ml of medium) were used to incubate the strains. During the incubation, bacterial cultures were sampled, followed by sonication and OD measurement. Growth curves from OD 600 values were fitted using the nonlinear regression function with the logistic model of SigmaPlot software (Systat Software Inc, CA, USA). The growth curve of one strain (B70) was not well fitted with this method. For that case, we manually calculated the specific growth rate from the raw data. The specific growth rate (μ), representing the maximum rate of change, was calculated by the change in OD 600, and the saturated cell density (maximum growth) was determined by its maximum value on the fitted growth curve.

### 2.7. Colony formation characteristic of isolated strains

The colony formation capability of the selected isolates obtained by NCFLC was further confirmed as follows. Identified OTUs were pure cultured in 5 ml of 1:10 diluted R2A broth at 25°C by shaking at 150 rpm. The strains were harvested at the stationary phase. Aliquots of the liquid cultures (5 μl) were inoculated on the agar plates using the same media used in the initial culture step and cultivated for 4 weeks. Then, it was confirmed whether colonies were present on the agar media. We conducted the colony formation test for all isolates (93 from soil samples) and for randomly selected OTUs isolated from the marine samples (132 OTUs in total).

### 2.8. DNA extraction and amplicon sequencing targeting the 16S rRNA gene

To compare the cultivated bacterial diversity with the microbial molecular signatures in the samples, 16S rRNA gene amplicon sequencing was performed. After transferring the samples to the laboratory, samples (1 g) were stored frozen at −20°C. Genomic DNA was extracted using the CTAB/SDS method, and DNA was diluted to 1 ng/μl. The 16S rRNA gene in the extract was amplified using the amplicon forward primer 341F (5′-CCTAYGGGRBGCASCAG-3′) and reverse primer 806R (5′-GGACTACNNGGGTATCTAAT-3′) (Yu et al., 2005) with Phusion^®^ High-Fidelity PCR Master Mix (New England Biolabs, USA) and then purified using a Qiagen Gel Extraction Kit (Qiagen, Germany). DNA was sequenced by Novogene (Beijing, China) using an Illumina HiSeq 2500 platform (Illumina, San Diego, CA, USA). Each read was assigned taxonomically using Quantitative Insights Into Microbial Ecology (QIIME) software (V1.7.0).

## 3. Results

### 3.1. Checking the efficiency of the new cultivation method

**Figure 2:**
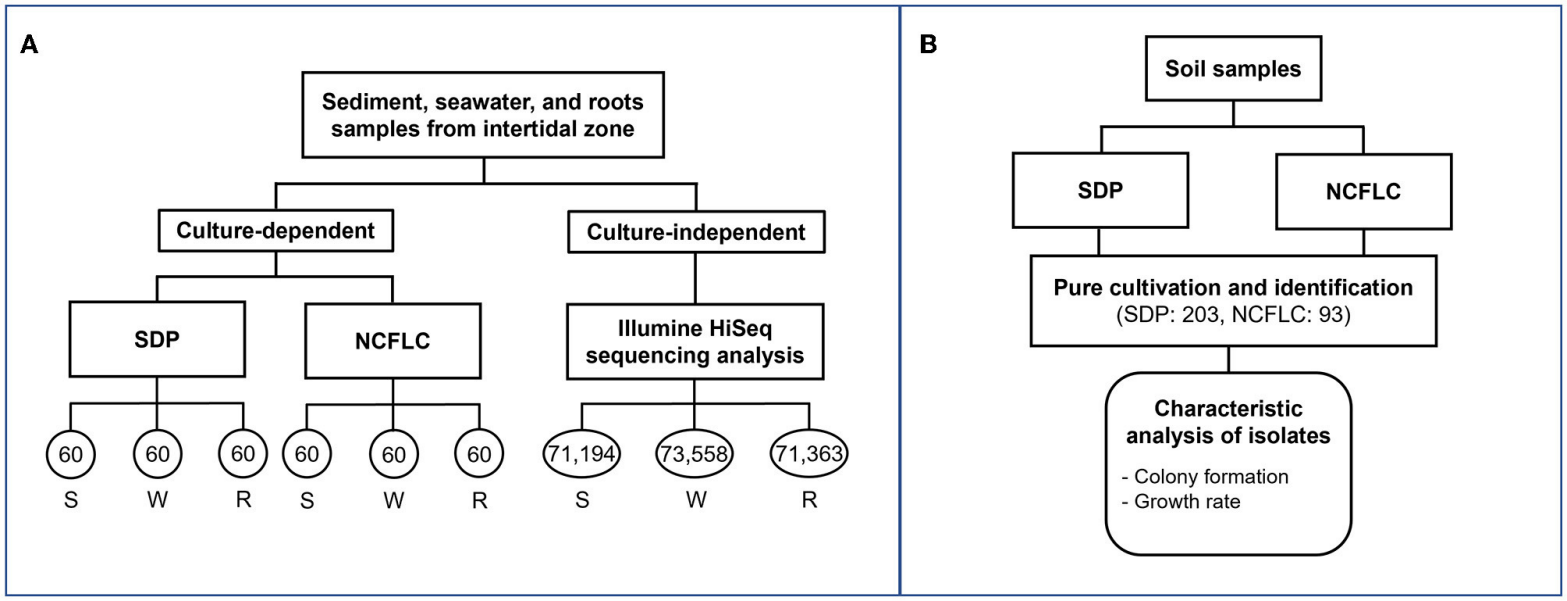
Flowchart of experiments with marine **(A)** and soil **(B)** samples. The numbers in each circle represent the number of isolates obtained from each culture-dependent and reads numbers Illumine HiSeq sequencing analysis of each sample **(A)**. The sample names, S, W, and R represent sediment, seawater, and marine plant roots, respectively **(A)**. The number of isolates obtained by the SDP and NCFLC cultivation methods was 203 and 93, respectively **(B)**. Characteristic analysis was performed for isolates from soil samples **(B)**.

A new cultivation procedure, Non-Colony-Forming Liquid Cultivation (NCFLC), was developed and applied to the marine and soil samples to isolate previously uncultured non-colony-forming microbes (Figure 2). The approach used in NCFLC included (1) dilution-to-extinction (DTE) cultivation and (2) a subsequent isolation procedure using agar plates to separate strains that do grow on solid media from those that do not (Figure 1).

We tested the NCFLC using three types of marine samples (sediment, S; seawater, W; marine plant roots, R) from the intertidal zone to confirm the efficiency of the new method, compared with the conventional standard direct plating (SDP) method (see experimental procedures section for detail; Figure 2A). A total of 360 bacterial isolates from the culture-dependent methods were identified (60 per each cultivation method and sample). The NCFLC method enabled the isolation of 98 species in total (defined as operational taxonomic units [OTUs] composed of 16S rRNA gene sequences sharing over 97% identity) from 10 taxonomic groups (*Acidobacteriota, Actinomycetota, Bacteroidota, Bacillota, Alpha-, Beta-, Gamma-* and *Epsilon- proteobacteria, Oligoflexia*, and *Verrucomicrobiota*; Supplementary Table S1), while 62 OTUs from 5 taxonomic groups (*Actinomycetota, Bacteroidota, Bacillota, Alpha-*, and *Gamma- proteobacteria*, Supplementary Table S2) were isolated by SDP. The NCFLC yielded 91 OTUs that were absent from the SDP collection, with an overlap of 7 OTUs (Supplementary Figure S1a). The collections at the species level differed depending on the cultivation methods and the sampling site (Supplementary Figures S1a, b).

**Figure 3:**
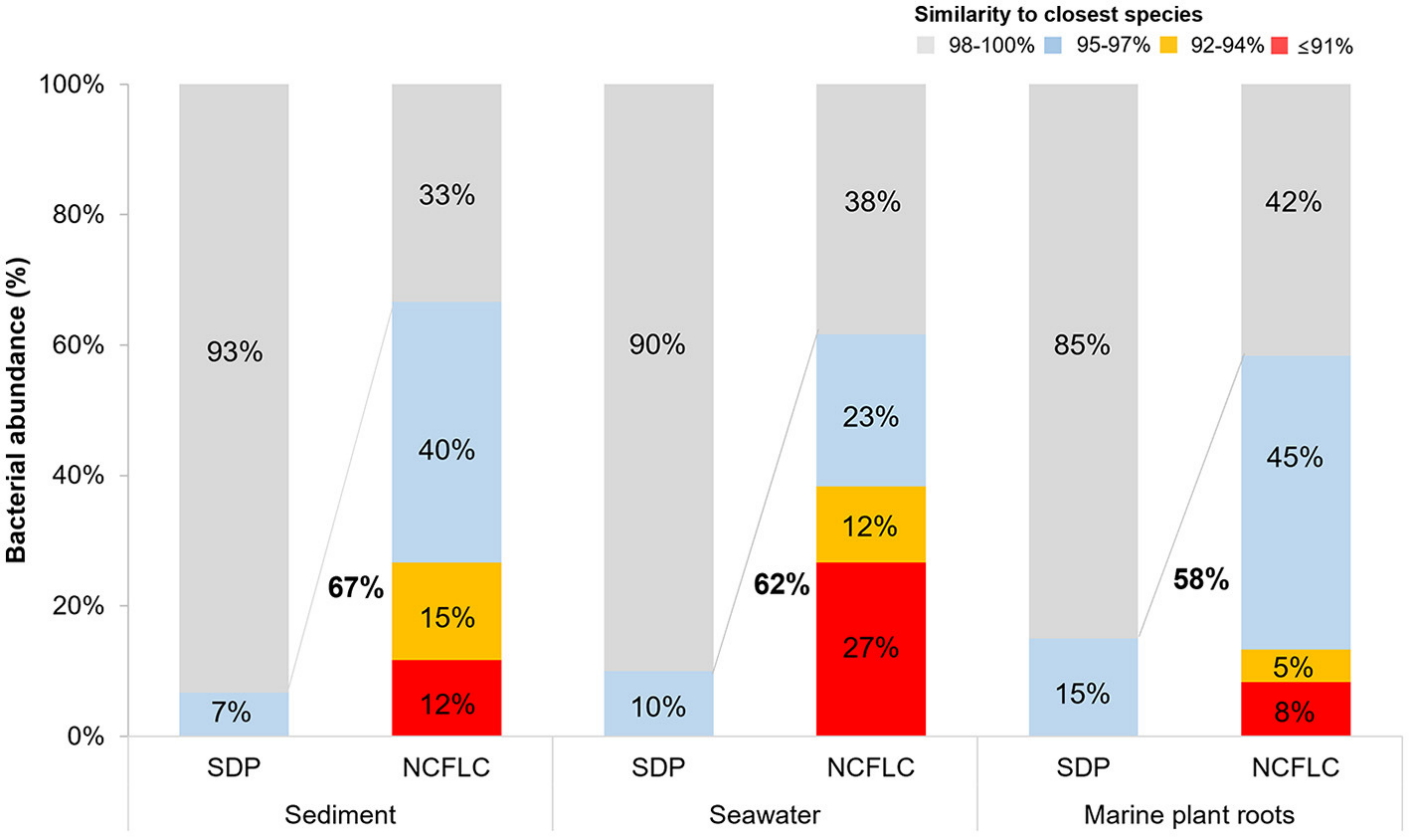
Comparison of the ratio of new species from the SDP and NCFLC methods for the three sampling sites in the intertidal zone. Novel species were defined as a strain with ≤97% 16S rRNA similarity to the closest known relative in the GenBank databases.

The proportion of potential novel species, defined as a strain with ≤97% 16S rRNA gene similarity to the closest known relative among the isolates in EzBioCloud, differed between the NCFLC and the SDP collections. Among the strains obtained by NCFLC from sediment, seawater, and marine plant roots samples, 67%, 62%, and 58% were novel species, respectively, while only 7%, 10%, and 15%, respectively, of the isolates from the SDP method were novel species (Figure 3).

**Figure 4:**
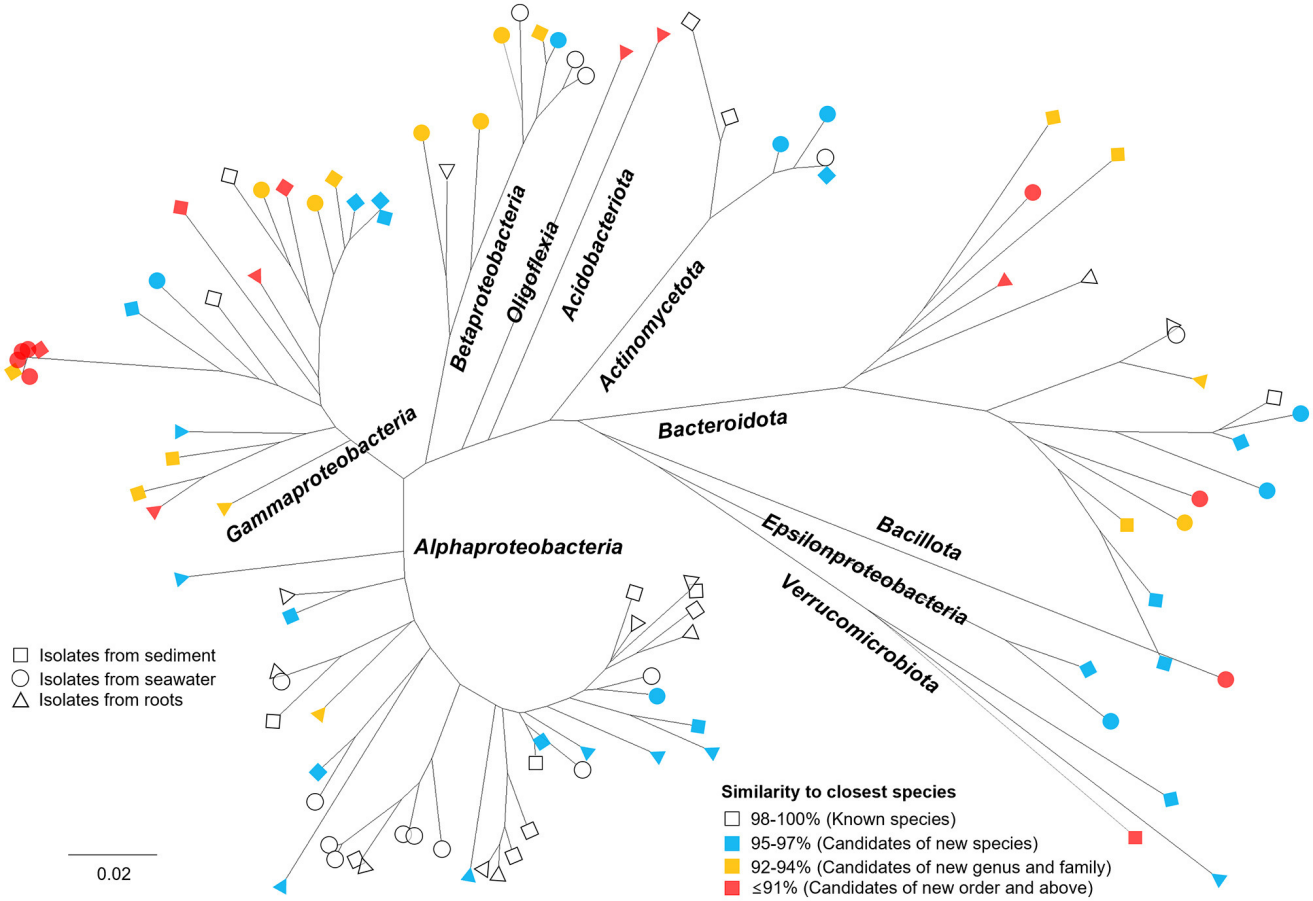
Phylogenetic tree showing the microbial diversity and novelty of all bacterial OTUs obtained using NCFLC. Trees are maximum-likelihood trees (fast bootstrap, 1,000 replicates) based on the 16S rRNA gene of isolates. The square, circle, and triangle represent isolates from sediment, seawater, and marine plant roots, respectively. The colors indicate the degree of similarity to the closest known species in GenBank.

The phylogenetic tree (Figure 4) of isolates cultivated by NCFLC exhibited extraordinary diversity, among which 62% were candidates as novel species. The new method also allowed for the isolation of several novel strains belonging to *Acidobacteriota, Epsilonproteobacteria, Oligoflexia*, and *Verrucomicrobiota*, which are known as fastidious types to cultivate. All 7 OTUs belonging to such rare groups show novelty at or above the species level.

We also used NCFLC to isolate bacteria from soil samples and compared the results with those from SDP to test if the new cultivation method was also effective for soil. A total of 203 and 93 bacterial isolates from SDP and NCFLC methods, respectively, were cultivated and identified (Figure 2B). The NCFLC enabled the isolation of 55 species (defined as above) from seven phyla (*Acidobacteriota, Actinomycetota, Armatimonadia, Bacillota, Alpha*-*, Beta*-, and *Gamma*-*proteobacteria*; Supplementary Table S3). Using SDP, 115 species from six phyla (*Actinomycetota, Bacteroidota, Bacillota, Alpha*-, *Beta*-, and *Gamma*-*proteobacteria*) were isolated (Supplementary Table S4). There were a few overlapping OTUs among the NCFLC and SDP isolates (Supplementary Tables S3, S4, Supplementary Figure S2), even though the inoculum used for each method was the same.

The proportion of novel species differed between the isolates from the two methods. Among the strains obtained by SDP, 9.4% of isolates (19 isolates belonging to 15 species) were novel species, while 21.5% (20 isolates belonging to 15 species) of the isolates from NCFLC were novel species (Supplementary Figure S3). Especially, the NCFLC method led to the cultivation of novel bacterial isolates belonging to *Armatimonadia* (closest species: *Armatimonas rosea*), known to be rare species in the identified isolates obtained by standard methods (Supplementary Figure S4).

### 3.2. Characteristic analysis of isolates

**Figure 5:**
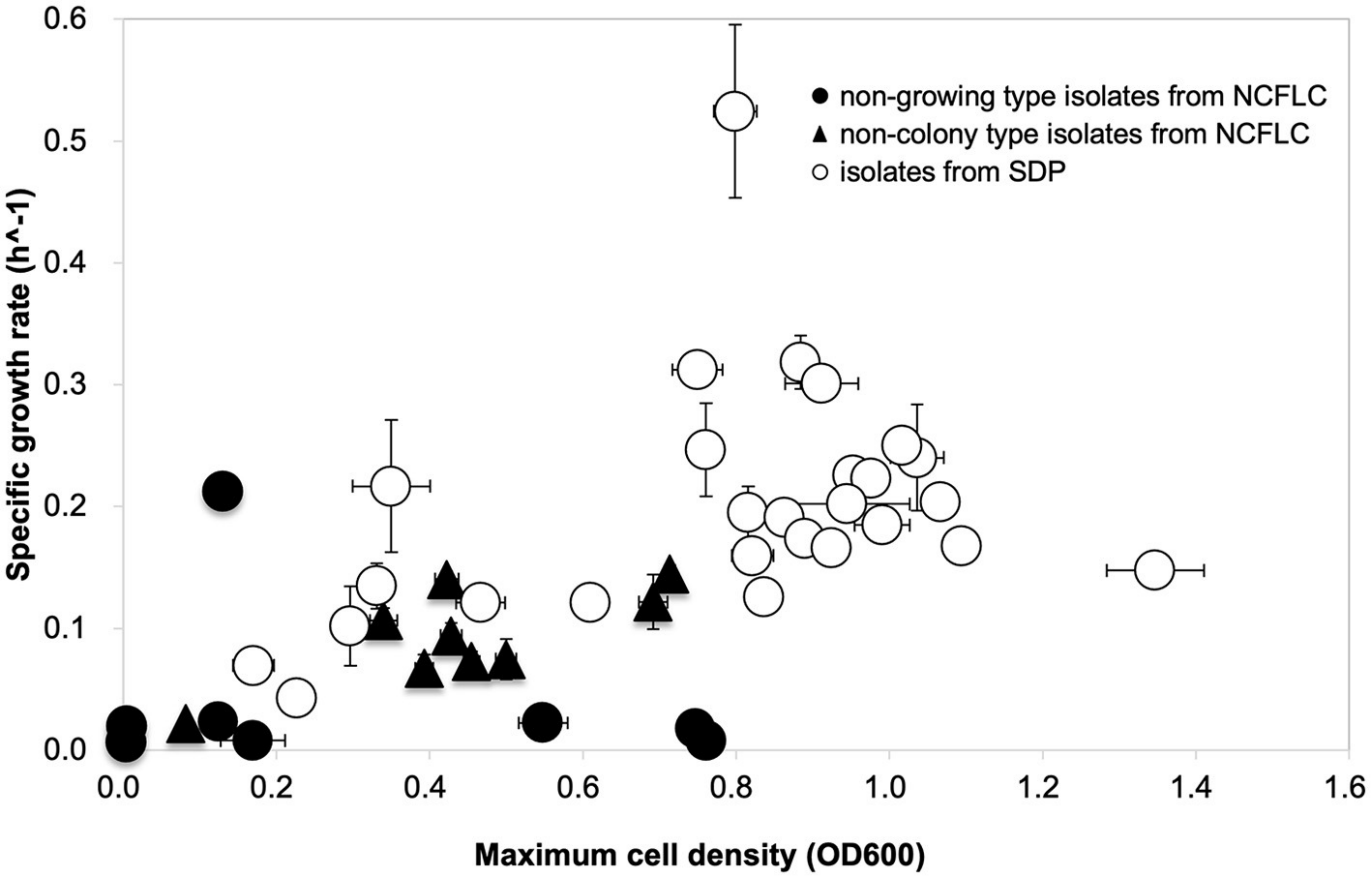
Growth curve comparison to characterize the physiological properties of the isolates. Approximately 17 strains (eight strains of non-growing type and nine strains of non-colony type) were obtained from NCFLC, and 27 strains from the SDP method were selected randomly for growth characteristics.

The specific growth rates and saturated cell density of the selected strains from the NCFLC and SDP methods are shown in Figure 5 (we hereafter define those selected strains as NCFLC and SDP strains, respectively). Most of the clusters for the NCFLC strains were distinct from the SDP strains, as the specific growth rates of most NCFLC strains (12/17, 70.6%) were significantly lower (<0.1 for growth rates) than those of the SDP, among which only two strains (2/27, 7.4%) showed lower than 0.1 for growth rates. Saturated cell densities significantly differed between liquid cultivation and agar plating (*t*-test; *p* < 0.05).

The colony formation of isolates obtained by NCFLC was confirmed on agar plates (Supplementary Figure S5). Among the selected 132 bacterial species obtained from NCFLC in marine samples, 34% (45/132) did not show any growth on agar plates (we have hereafter defined those strains as non-colony type on agar plates), 52% (68/132) did not form any ordinary-sized colonies on agar plates (we have hereafter defined those strains as micro-colony type with a diameter smaller than 100 μm), and 14% (19/132) formed normal-sized (diameter larger than 100 μm) colonies (Supplementary Figure S5a). Among 55 OTUs from soil samples, 9 (16%) were non-colony type, and all other tested isolates were micro-colony type (Supplementary Figure S5b). The proportion of novel species among normal-sized colonies was 45%, whereas that of non-colony type and micro-colony type were 100 and 72%, respectively.

### 3.3. Bacterial community composition determined by the culture-independent method

**Figure 6:**
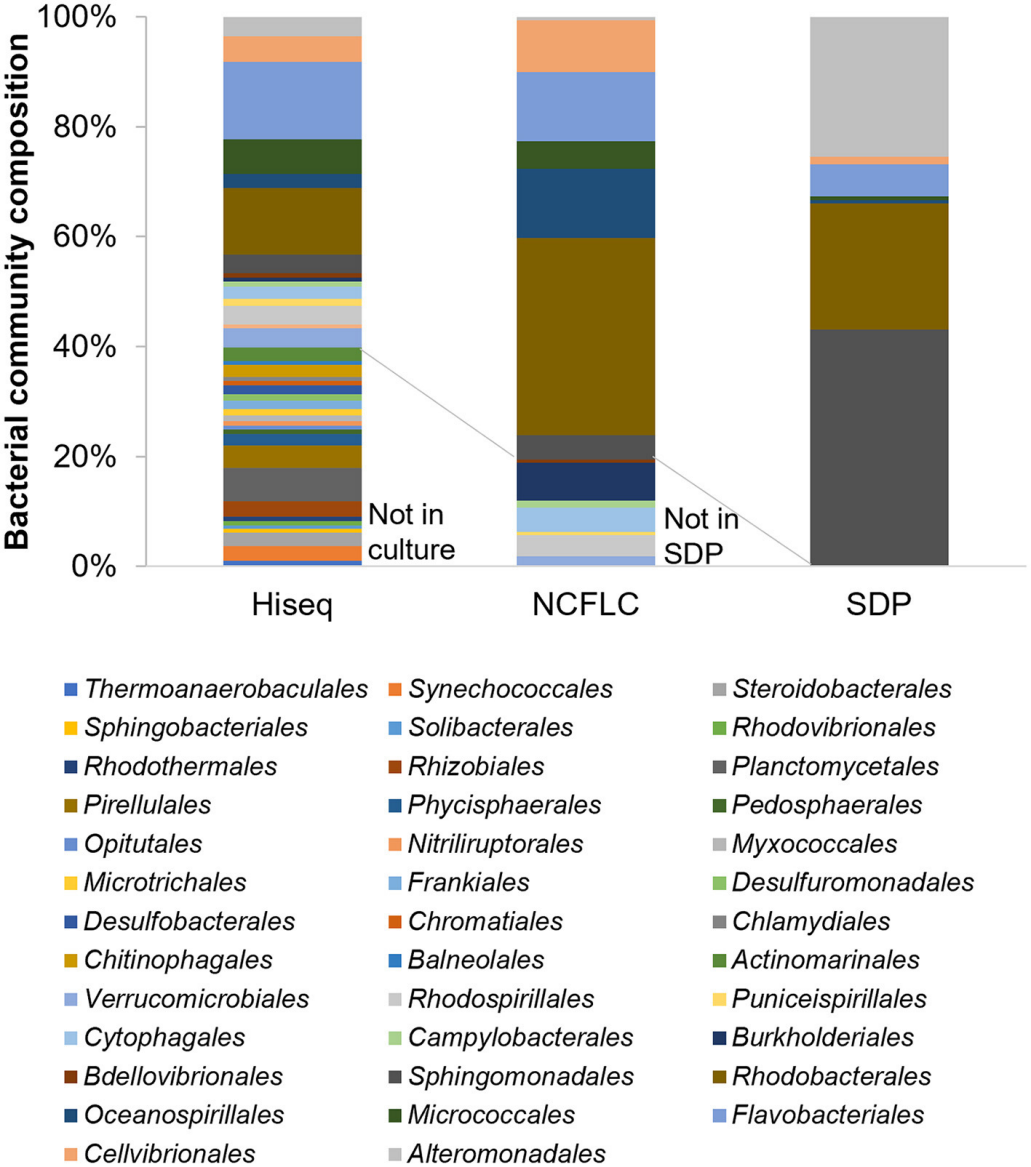
Bar graph showing the bacterial community composition at the order level for the samples by HiSeq sequencing analysis (with a cut-off of < 0.02%) and the isolates obtained by the NCFLC and SDP methods.

The composition of the microbial community in the three types of samples—sediment (S), seawater (W), and marine plant roots (R)—was analyzed by Illumina HiSeq sequencing based on the 16S rRNA gene. A total of 71,194, 73,558, and 71,363 reads with a median length of 250 base pairs (bp; V3–V4~433–682 bp) assigned to 3,612, 1,578, and 2,800 OTUs, respectively, was obtained from each sample. In total, eight major phyla (cut off <1.0%), *Acidobacteriota, Actinomycetota, Bacteroidota, Cyanobacteria, Patescibacteria, Planctomycetota, Pseudomonadota*, and *Verrucomicrobiota*, were detected from the samples (Supplementary Figure S6). The isolates from the culture-dependent methods belonged to five groups, *Acidobacteriota, Actinomycetota, Bacteroidota, Pseudomonadota*, and *Verrucomicrobiota*. However, *Cyanobacteria, Patescibacteria*, and *Planctomycetota* did not appear in isolates. The most abundant phylum was *Pseudomonadota* in all samples, similar to the results obtained by the culture-dependent methods (Supplementary Tables S1, S2). We also analyzed the 16S rRNA sequences from Illumina-HiSeq sequencing at the order and species levels and compared the results with those of culture-dependent approaches. A total of 38 major orders were identified (with a cutoff of < 0.1%) in the environmental samples. Among those, the isolates from the NCFLC method belonged to 14 orders, while the isolates from the SDP method belonged to only seven orders (Figure 6). At the species level, a total of 581 major species were identified (with a cutoff of < 0.1%) in the environmental samples. Among those, the isolates from NCFLC belonged to 98 species, while the isolates from SDP belonged to 62 species (data not shown).

## 4. Discussion

The majority of microbes do not form colonies on standard agar media used for microbial cultivation (Amann et al., 1995; Rinke et al., 2013). To cultivate these previously uncultured microbes, the NCFLC method was developed.

To target strains that can only be cultured in a liquid medium, new procedures to separate these strains from other bacterial types are essential. The dilution-to-extinction (DTE) cultivation method was employed as the first step to distribute bacterial cells from the samples using liquid media into 96-well cell culture plates (Connon and Giovannoni, 2002; Rappé et al., 2002). A wide range of sample dilutions were inoculated to achieve the optimal dilution for further subculturing steps.

In our experience, dilutions allowing 10%−50% of wells showing bacterial growth could generate a substantial proportion of pure cultures and were sufficient to obtain clonal cultures for further sub-cultivation (Yu et al., 2005). Therefore, we used concentrations of sample dilutions that allowed ~50% of the wells to show bacterial growth and to be used for subculturing. Among these grown liquid cultures in selected dilutions, ~3% of the cultures were the bacterial type that only grew in liquid medium (but did not grow on a solid agar plate), which was ~1% of total inoculated wells (data not shown). We usually used 36 plates (96 multi-well) for each trial (3,456 wells), which produced 30–40 candidate isolates that showed growth only in liquid media in the selection step. The ratio of pure cultures confirmed by 16S rRNA gene sequencing after transfer from 96 multi-well plates to the liquid media was 73% ± 12% (data not shown). This approach clearly shows that a sufficient number of the targeted bacterial-type isolates can be obtained using the procedures in NCFLC.

We applied the NCFLC method to several types of marine samples to verify the efficiency of the new tool for isolating new microbial resources (Figure 3). The NCFLC produced a number of previously uncultured microbes and was more efficient than any previous studies (Rappé and Giovannoni, 2003; Pham and Kim, 2012; Lewis et al., 2021). Particularly, the NCFLC method led to the isolation of bacterial species from rare phyla in the identified isolates, including *Acidobacteriota, Epsilonproteobacteria, Oligoflexia*, and *Verrucomicrobiota*, that were not cultivated by the SDP method (Figure 4). All isolates belonging to those groups from NCFLC were previously uncultured and undescribed bacterial species (Supplementary Table S1, Figure 4).

Comparing the bacterial composition of the 16S rRNA gene from the culture-independent method and that of the isolates indicated that many bacterial groups that could not be cultivated using SDP could be isolated using the NCFLC method. The NCFLC led to the isolation of several bacterial groups at the order level (seven orders) that were present in the Illumina HiSeq sequencing data, but they were not cultivated using SDP (Figure 6). It is clear that NCFLC produced many isolates that were absent in standard agar-based methods. This indicates that some bacterial species known as “uncultured” are not among the identified isolates because those species are not capable of growing on solid plates but may also be missed in standard liquid cultivation studies without the selection process we developed. In contrast, we noted an absence of several orders (24 orders) in isolates compared with a culture-independent method, even though a high proportion of these groups were found in the samples (Figure 6). These microbes likely require other specific growth conditions not met in either NCFLC or SDP.

In the case of soil samples, NCFLC also led to the isolation of a different set of bacterial species that were more novel than that obtained using SDP (Supplementary Figure S3). Approximately 71% (11/55) of isolated OTUs using NCFLC did not appear in isolates from SDP (Supplementary Tables S3, S4, Supplementary Figure S2), even though the inoculum used for both methods was the same. This is not surprising considering that we targeted only isolates that could not grow on solid agar plates during the selection step (Figure 1). In addition, some microbes can only grow in liquid media or have difficulty forming ordinary-sized colonies on an agar medium. Those species would be more likely to be undescribed (unknown) species than isolates grown on conventional agar medium since most of the microbial species in identified isolates (e.g., NCBI) have been cultivated and isolated by agar-based cultivation methods in the earliest experiments in microbial cultivation (Lok, 2015). The NCFLC method successfully isolated a novel microbe, that is, strain B70 (closest species: *A. rosea*; Supplementary Figure S4). Phylogenetic analysis based on 16S rRNA gene sequence (almost full length, >1,300 bp) clearly showed that the strain B70 belongs to the rare phylum, *Armatimonadia*, which was tentatively classified as candidate phylum OP10. Only a few strains belonging to the *Armatimonadia* have been isolated to date (Lee et al., 2011; Tamaki et al., 2011; Im et al., 2012).

We also characterized the physiological properties of the isolates from each method, attempting to clarify the mechanisms operating in the NCFLC method. Comparing growth curves among isolates from NCFLC and SDP, we found that specific growth rates and saturated cell densities (carrying capacity) of NCFLC isolates were much lower than those of SDP isolates (Figure 5). This suggests that the NCFLC strains were not as competitive as pure cultures on agar plates and in liquid cultivation. Therefore, separating each bacterial species with DTE cultivation with an appropriate dilution is an essential step to isolate this bacterial type. Furthermore, the presence of favorable conditions for slow-growing bacteria does appear to be related to the mechanisms allowing the isolation of unique bacterial groups because we found that no isolates from NCFLC could grow or had difficulty in forming ordinary-sized colonies on agar plates even after they were purified (Supplementary Figure S5). Previous studies have successfully demonstrated that liquid cultivation using a continuous-flow bioreactor led to isolating previously uncultured species, especially bacterial types inhibited by their own growth. Thus, this type formed significantly smaller-sized colonies on agar media than strains from SDP during the sub-cultivation step (Jung et al., 2022). Therefore, the growth of some isolates from NCFLC in this study was also likely inhibited on agar plates because of sensitivity to their own metabolic compounds. Consequently, they cannot grow into high cell densities on agar media and in liquid culture.

Additional factors to be considered in this context are that the conditions in the laboratory cultivation (nutrient concentrations, pH, gas composition, etc.) were not perfectly fit for cultivating certain bacterial species. The results from the colony formation test showed that most of the isolates obtained using NCFLC were micro-colony types (Supplementary Figure S5). These isolates had difficulty forming ordinary-sized colonies. According to previous studies, microcolonies are microscopic bacterial colonies grown under growth-limiting conditions (Anderl et al., 2003; Walters et al., 2003; Borriello et al., 2004). Other studies have shown that colony formation is affected by several factors, such as the presence or absence of certain nutrients, gas concentrations, and carbon concentrations (Simu et al., 2005; D'Souza et al., 2021). Therefore, some bacteria could not grow well on agar plates and were undetected during the sub-cultivation step (Figure 1). However, the conditions for their growth in liquid culture were adequate for detection. In this study, we confirmed that NCFLC can produce unique isolates that SDP cannot isolate. Thus, we are confident that the NCFLC method is efficient for isolating new microbial species.

In summary, we developed a novel liquid cultivation procedure to selectively isolate the bacterial type that is difficult to grow on solid agar medium and employed NCFLC to isolate previously uncultured bacterial species from environmental samples. The NCFLC method allowed the isolation of numerous novel species (a total of 63 species in marine samples) that could not be achieved using a conventional cultivation method. Furthermore, by comparing the physiological properties of isolates from different approaches (NCFLC and SDP), the findings here confirm that isolates obtained by the new liquid cultivation method have (1) difficulty growing on agar media and (2) lower growth rates and saturated cell densities than isolates from the SDP method. Our results clearly indicate that the NCFLC method can selectively cultivate novel bacterial species better than conventional methods.

Together with the results from our previous studies (Jung et al., 2021a,b), improvements in techniques to access previously uncultured microbes are well underway. Continued advances in developing cultivation methods that investigate the causal mechanisms of microbial uncultivability, including the NCFLC method described here, will allow new insights into accessing the remaining part of the microbial dark matter.

## Data availability statement

The datasets presented in this study can be found in online repositories. The names of the repository/repositories and accession number(s) can be found in the article/Supplementary material.

## Author contributions

E-YS and DJ conceived, investigated, carried out most experiments, and wrote the original draft. SE, JO, YA, and SH provided critical feedback on the research and reviewed and edited this manuscript. WZ, HB, AY, MH, YN, and SK helped in the visualization and investigation of the research analysis. YA and SH were in charge of research administration and supervision of this research and took responsibility for the accuracy and integrity of all data. All authors contributed to the article and approved the submitted version.
