# Supplementary material for: A targeted liquid cultivation method for previously uncultured non-colony forming microbes
Source: Front Microbiol. 2023 Jun 9;14:1194466. doi: 10.3389/fmicb.2023.1194466 (PMC10288195; doi:10.3389/fmicb.2023.1194466)
Supplement: Supplementary file 5 [file Data_Sheet_1.PDF]

# *Supplementary Material*

## 1 Supplementary Figures and Tables

### 1.1 Supplementary Figures

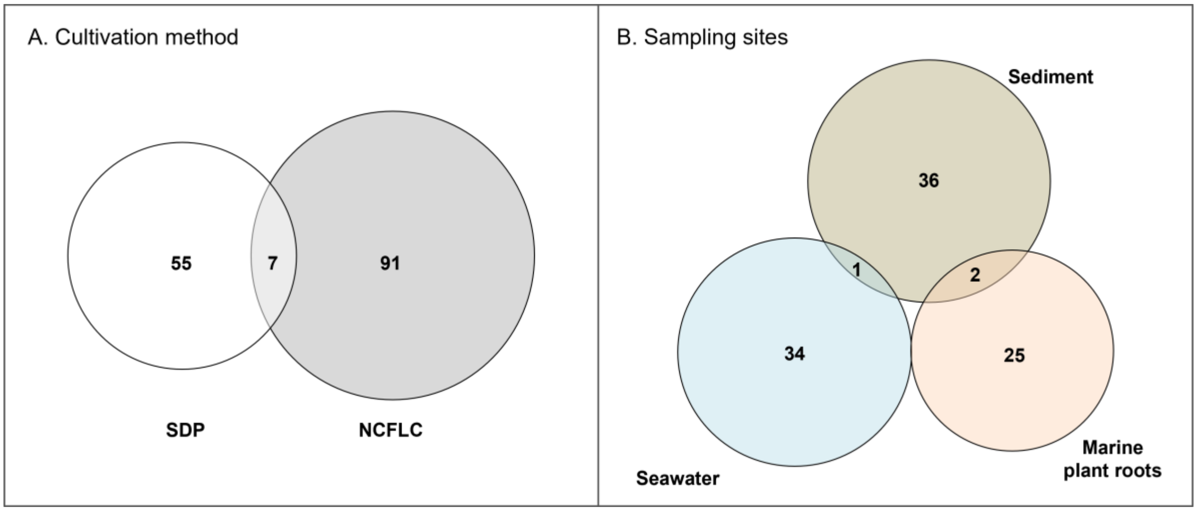


**Supplementary Figure 1.** Venn diagrams of the microbial species (a) between the cultivation methods (SDP and NCFLC) and (b) three types of marine samples (sea sediment, seawater, and marine plant roots). The number of obtained isolates from SDP and NCFLC were each 180 and, from three types of marine samples were 60, respectively.

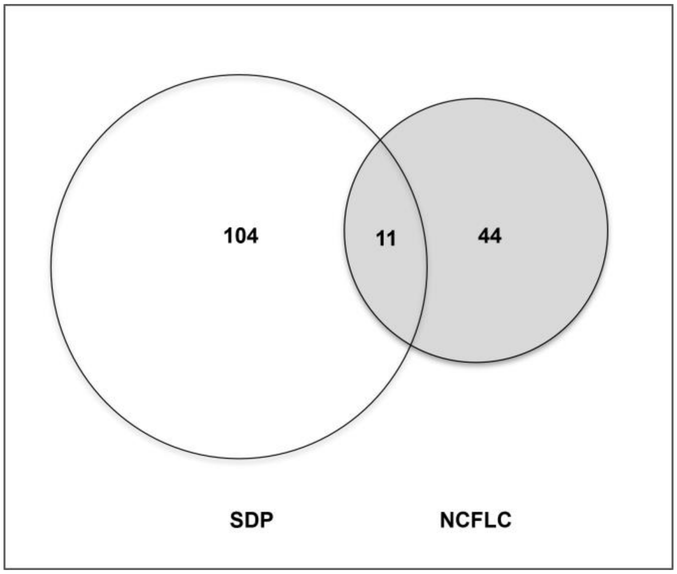


**Supplementary Figure 2.** Venn diagrams of the microbial species between the cultivation methods of soil samples. The number of isolates from SDP and NCFLC method were 203 and 93 respectively.

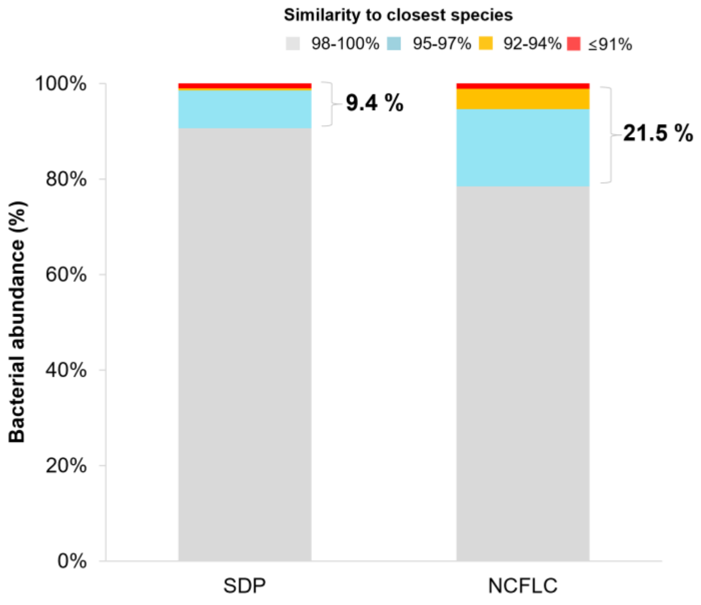


**Supplementary Figure 3.** Comparison of the ratio of new species from SDP and NCFLC methods for soil sample. Novel species were defined as a strain with ≤97% 16S rRNA similarity to the closest known relative in GenBank databases.

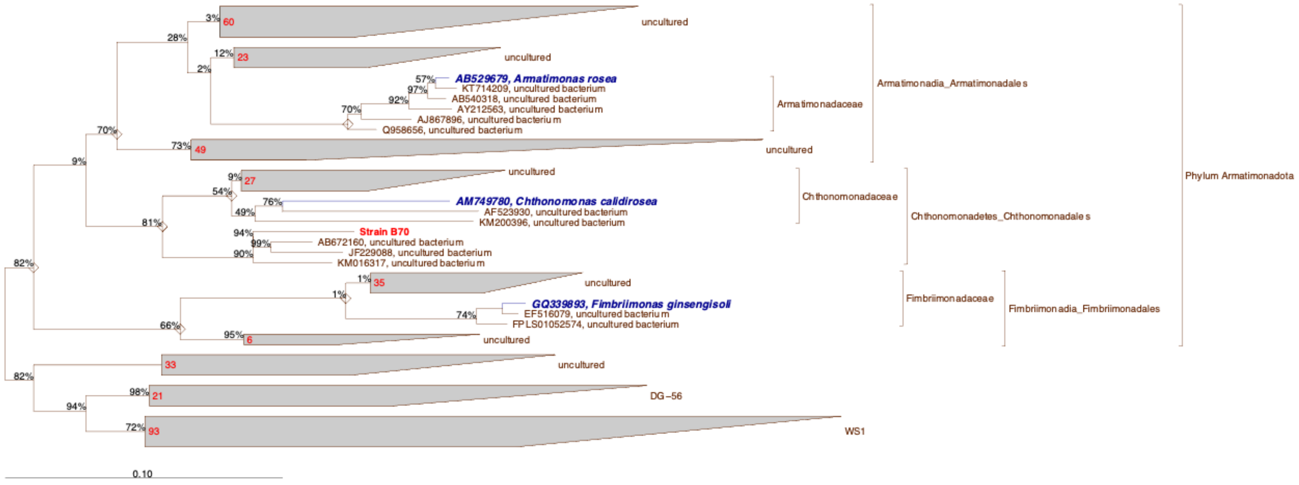


**Supplementary Figure 4.** Phylogenetic tree showing the novel bacterial isolates belonging to *Armatimonadia* from NCFLC in soil samples.

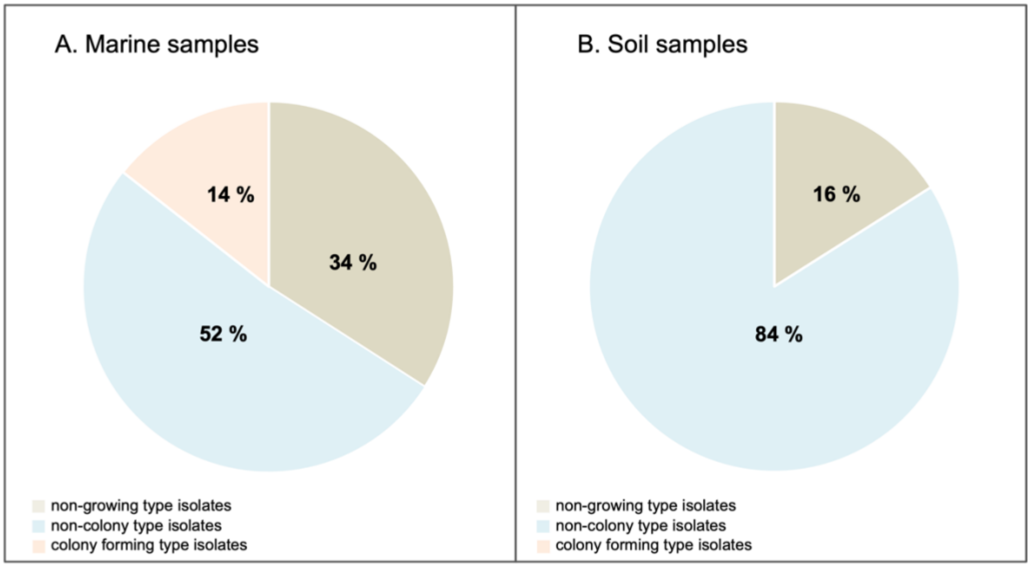


**Supplementary Figure 5.** Re-confirmation of colony formation of isolates obtained from NCFLC in (a) marine samples and (b) soil samples. All isolates (93 isolates belong to 55 species) from the soil sample and randomly selected 132 species in marine samples were tested.

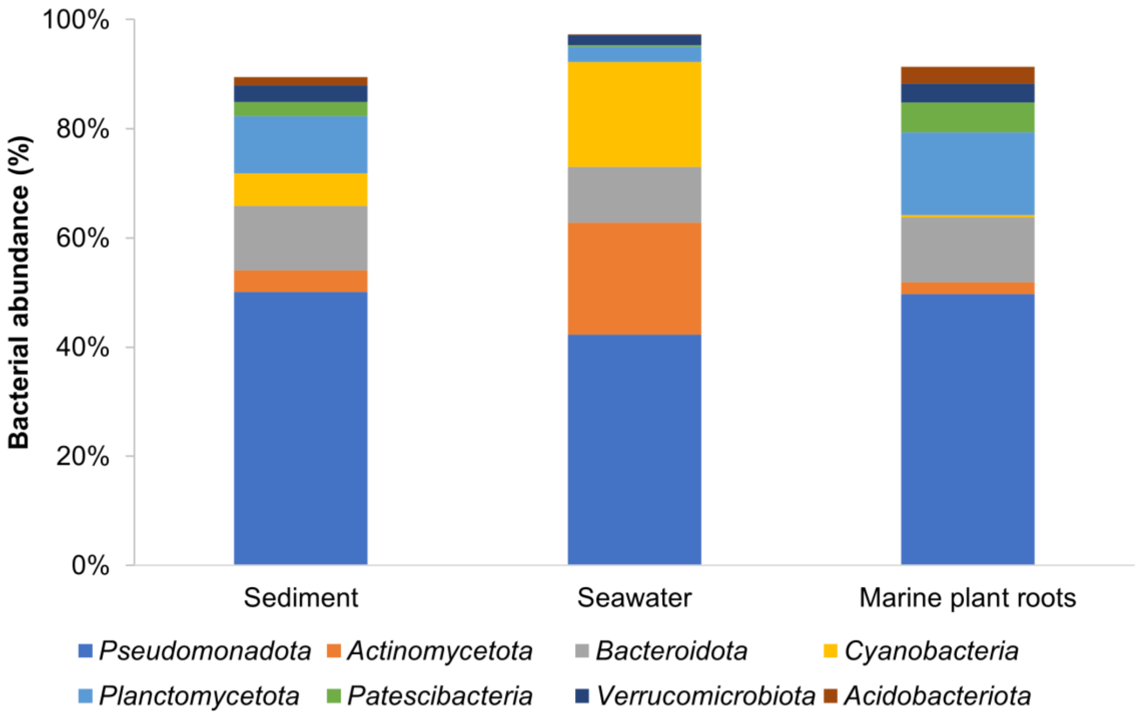


**Supplementary Figure 6.** Bacterial composition at the phylum level for three types of samples in marine sample by HiSeq sequencing analysis (with a cut-off <1.0%).

## 1.2 Supplementary Tables

**Supplementary table 1.** Phylogenetic affiliations of isolates from sediment, seawater and marine plant roots in marine with the NCFLC method based on 16S rRNA gene sequences.

**Supplementary table 2.** Phylogenetic affiliations of isolates from sediment, seawater and marine plant roots in marine with the SDP method based on 16S rRNA gene sequences.

**Supplementary table 3.** Phylogenetic affiliations of isolates from soil samples with the NCFLC method based on 16S rRNA gene sequences.

**Supplementary table 4.** Phylogenetic affiliations of isolates from soil samples with the SDP method based on 16S rRNA gene sequences.
